# Depressive and Anxiety Symptoms among Pediatric In-Patients with Dengue Fever: A Case-Control Study

**DOI:** 10.3390/ijerph17010099

**Published:** 2019-12-21

**Authors:** Von Ralph Dane M. Herbuela, Ferdinand S. de Guzman, Girly D. Sobrepeña, Andrew Benedict F. Claudio, Angelica Cecilia V. Tomas, Carmina M. Arriola-delos Reyes, Rachele A. Regalado, Mariama M. Teodoro, Kozo Watanabe

**Affiliations:** 1 Graduate School of Science and Engineering, Ehime University, Bunkyo-cho 3, Matsuyama 790-8577, Japan; herbuelavonralphdane@gmail.com; 2 Department of Family and Community Medicine, Infectious Diseases and Tropical Medicine, San Lazaro Hospital, Manila 1003, Philippines; fdgzm06@gmail.com; 3 Pediatrics Department, Quezon City General Hospital, Quezon City 1106, Philippines; sobrepenagirly@yahoo.com; 4 Office of Nursing Service, Pasay City General Hospital, Pasay City 1300, Philippines; tomriddle_07@yahoo.com; 5 Department of Pediatrics, College of Medicine, University of the Philippines, Manila 1000, Philippines; anggetomas@gmail.com (A.C.V.T.); minettedr@yahoo.com (C.M.A.-d.R.); 6 Guidance Department, University of Santo Tomas–Angelicum College, Quezon City 1114, Philippines; cheleregalado@gmail.com; 7 Counseling and Educational Psychology Department, De La Salle University, Manila 1004, Philippines; mariamateodoro@gmail.com; 8 Biological Control Research Unit, Center for Natural Science and Environmental Research, De La Salle University, Manila 1004, Philippines

**Keywords:** dengue fever, depression, anxiety, pediatric psychiatry

## Abstract

Background: Psychiatric symptoms have been reported in adult patients with dengue fever (DF); however, information on pediatric patients remains inadequate. We sought to identify the prevalence and predictors of depressive and anxiety symptoms and identify other psychiatric symptoms among pediatric patients with DF. This case-control study involved pediatric in-patients (*n* = 225) who had clinical or serologic-confirmed DF and healthy school-based controls (*n* = 260). Participants completed the Revised Child Anxiety and Depression Scale (RCADS). Results: The prevalence of depressive (13.3%) and anxiety (34.2%) symptoms among pediatric patients with DF was significantly (*p* < 0.001) higher than that among controls (3.5% and 16.2%, respectively). Multiple linear regression analysis found that age, family history of DF, ≤2 days of hospitalization, myalgia, and arthralgia were predictors of increased depressive and anxiety symptoms among the patients. Further, 26.7% of pediatric patients reported irritability, agitation, visual hallucinations, and aggressiveness. Conclusion: Pediatric patients present depressive and anxiety symptoms whose levels were associated with social and clinical factors. However, whether these symptoms are present only during the infection or may still persist after recovery or are brought by children’s adverse reactions to hospitalization are unknown, and thus, further studies are needed.

## 1. Introduction

As the world’s most rapidly spreading mosquito-borne disease, dengue fever (DF) causes approximately 50 million cases a year, putting an estimated 2.5 billion people at risk, especially children [1]. Its incidence has increased by 30 times in the last five decades [2] and is now prevalent in 128 countries [3]. It presents flu-like symptoms that include high-grade fever accompanied by headache, myalgia and arthralgia, nausea and vomiting, intense abdominal pain, rash, retro-/peri-orbital pain, bleeding, and low platelet count (thrombocytopenia), which can lead to acute organ failure, cardiomyopathy, encephalitis, profound shock, and death [1]. Recently, the number of studies reporting this virus to be neurovirulent has increased [4], associating it with neurological complications in patients with DF [5].

The most prevalent neurological disorder occurring during DF is encephalopathy [6]. Case study reports found that adult patients with DF exhibit delusions with auditory and visual hallucinations, agitation and psychotic symptoms and fears, agitation, irritable affect, psychosis, mania, and catatonia [7,8,9,10,11,12,13,14]. The link between these symptoms and DF infection have been thought to be the result of metabolic disturbances, direct tissue lesion, intercranial hemorrhage, cerebral edema and anoxia and hyponatremia [7,11,12]. Among the identified encephalopathies, depressive and anxiety symptoms were the most studied among adult patients with DF [15,16,17,18]. The prevalence of borderline and clinical depression and anxiety among adult patients with DF ranges from 60% to 81% [16,17]. During the acute phase, the majority (90%) of patients exhibit thanatophobia or fear of death (90%), and during the recovery phase (1 week after onset of DF), more than half (55%) of patients develop fear of mosquitoes (55%) [15,18]. Most importantly, the severity of DF symptoms such as fever, headache, myalgia, arthralgia, retro-/peri-orbital pain, and thrombocytopenia positively correlate with depression and anxiety [16] and increased levels of proinflammatory cytokines such as Interleukin 4/6 and tumor necrosis factor (TNF)-alpha, and platelet brain-derived neurotrophic factors (BDNF) account for the presence of depressive and anxiety symptoms among adult patients with DF [16].

Depression and anxiety have been investigated in children with fatigue syndrome, fibromyalgia, migraine/tension headache [19], chronic abdominal pain [20], juvenile idiopathic arthritis [21], cancer [22], and human immunodeficiency virus (HIV) [23], yet information among pediatric patients with DF remains inadequate. One prospective study provides information on neurologic manifestations of DF in children. Pancharoen and Thisyakorn [24] reported that 80 of 1493 (5.4%) Thai in-patient children (3 months to 14 years old), who had serologically confirmed DF diagnosis, exhibited seizures and encephalopathy-like depressed sensorium and mental confusion during the febrile (acute) stage of DF based on recorded medical charts. However, studies that prospectively use a self-report screening tool to detect depressive and anxiety symptoms [25], which has a high acceptance and response rate (99%) and a low refusal rate (1%) among in- and out-patients [26], are lacking. To date, the study done by Mushtaq and Zahir [27] is the only known study, to our knowledge, that has used a self-report screening tool to measure depression, anxiety, and stress among pediatric patients with DF and investigated its relationship with self-efficacy. The study used the Depression Anxiety and Stress Scale (DASS) to measure depression, anxiety, and stress and the General Self-Efficacy Scale (GSES) to measure self-efficacy among the participants [27]. They found out that self-efficacy has a negative correlation with depression, anxiety, and stress; thus, developing self-efficacy among the patients is deemed necessary [27]. Although this study adds to the emerging topics on the presence of depressive and anxiety symptoms among pediatric patients with DF, there is still a pressing need to conduct more studies to measure the impact of DF infection on the mental health of pediatric patients.

Therefore, this study aimed to estimate the prevalence of depressive and anxiety symptoms among pediatric in-patients with DF and compare it with that among healthy school-based controls. We also sought to explore the predictors of these symptoms and to identify other self/parent-reported psychiatric manifestations that occur during the infection. We hypothesized that the prevalence of depressive or anxiety symptoms among pediatric patients with DF would be higher than that among controls, and predictors would include pain-related DF symptoms including headache, myalgia, arthralgia, retro-/peri-orbital pain, and abdominal pain suggesting a causal link between depressive, anxiety, and other psychiatric symptoms and DF infection.

## 2. Materials and Methods

### 2.1. Study and Sampling Design

This case-control study involved pediatric patients (cases) admitted at three public tertiary (>100 beds) hospitals in Metro Manila, Philippines, from July to November 2017, during the high transmission of DF cases. Metro Manila was chosen due to an increase in the number of DF cases (15.5% increase compared with previous year [28]) from 1 January to 6 May (morbidity week, 1–18), which was one of the highest rates in the country in 2017 [29]. Simultaneously, healthy grade 3 to 12 students, whose ages were similar with the cases, 8 to 17 years old, were also recruited to serve as controls. Since some of the patients with DF could not answer the test because of sickness, we asked their parents to answer the parent version of the screening tool. Thus, we also recruited parents of children whose age and grade level matched those of the patients with DF, as controls. We used the 1:1 ratio (one case patient/one control) with an assumed odds ratio of ≥2, power (1-β) of 0.80, 0.05 significance level, Zα = 1.96 [30], however, we were able to recruit a large number of controls. We failed to match them with the cases to control the confounding effects of age, gender, and grade level because availability of controls with matching criteria with the cases was limited during collection. While we also acknowledge the importance of obtaining controls in the same hospitals as the cases, we opted to sample controls in schools, who, like the cases, were students. We assumed that recruiting students as controls would increase our chance to sample healthy (no DF (like the cases) or any psychiatric and/or medical conditions) controls to investigate the true association between depressive and anxiety symptoms and DF infection.

### 2.2. Participant Inclusion and Exclusion Criteria

Participants were selected based on the pre-determined inclusion and exclusion criteria, availability, willingness to communicate their experiences and participate in the study [31,32,33]. A semi-structured interview was conducted among pediatric in-patients with serologically (laboratory) confirmed or clinically diagnosed DF and healthy school-based controls with no current or existing signs and clinical symptoms or diagnosis of DF. Recruitment of the patients and controls was also based on age (age 8–17 years) and grade level (grades 3 to 12) criteria of the Revised Child Anxiety and Depression Scale (RCADS-25), a screening tool for depressive and anxiety symptoms. Eligible participants had no history and/or existing diagnosis of psychiatric and/or medical conditions for which they received medical advice or treatment prior to the interview. Patients with life-threatening comorbidities and controls who were not able to comply with consent procedures were excluded.

### 2.3. Ethical Considerations and Data Collection Procedures

Our study was approved by the Research Ethics and Review Unit of San Lazaro Hospital, Research Ethics and Technical Committee of Pasay City General Hospital and Planning, Development, Education, and Research office of Quezon City General Hospital (ethics approval numbers: RERU-SLH 2017-016 E and PGH-RETC-01-0-091417001DENGUE). It complied with the ethics guidelines of the Declaration of Helsinki [34], International Council for Harmonization-Good Clinical Practice (ICH-GCP) guidelines [35], and National Ethical Guidelines for Health Research [36]. We also followed the reporting guidelines of case-control studies required by the STrengthening the Reporting of OBservational Studies in Epidemiology (STROBE) statement checklist in writing this manuscript (File S1).

All the participants (especially the patients, and their parents, legally authorized representatives (LARs) or caregivers) gave their consent before participating in this study. They were asked to read and sign an informed consent form (and assent form for children below 18 years old) in Filipino and were informed that participation in the study was voluntary and they may stop their participation at any time. Interviews were rescheduled for patients who could not participate due to sickness and severity of symptoms (e.g., intensive care unit confinement for severe dengue patients). In some cases, a parent, LAR or caregiver was asked to complete the forms and the RCADS-25-parent version for their children. Recruitment and interviews of patients were done by a primary investigator who underwent ICH-GCP guidelines training and with supervision from co-investigators (e.g., physicians, nurses) at each hospital. A psychologist and a guidance counselor recruited and interviewed the controls. A semi-structured interview (consistent pre-determined instructions, questions, and standardized screening tool) was done among all the participants to keep the data free from inconsistencies, incomplete responses, and biases. All forms and tests with confidential information of the participants were coded. Participants were informed about their scores on the tests, and a consent for further psychiatric assessment was solicited from those whose scores suggested a need for clinical intervention during follow-up and those who reported other psychiatric manifestations during the onset of the infection.

### 2.4. Forms and Measures

#### 2.4.1. Explanatory Variables

Socio-demographic profile, clinical parameters and symptoms: Socio-demographic profile included age, civil status, gender, educational attainment, occupation, family’s monthly income. and living arrangements. For age, we adjusted the age–disease association data (simple clustering method: k-means) which closely matched the accepted Medical Subject Heading (MeSH) ranges, into three age groups: 8–11, 12–14, and 15–17 based on the age criteria of RCADS-25 [37]. Income of the participants was identified by the help of their parents, guardian or LAR using the clusters from the indicative range of monthly family incomes (for a family of 5) in 2015 and 2017 [38]. However, we decided to have two clusters (above and below 10,000 pesos) as most of the patients had income below 10,000 pesos.

We also collected clinical information from the patients that included admitting diagnosis information, self and family history of DF, serologic test results (non-structural protein 1 antigen (NS1Ag) and blot test immunoglobin G antibody (IgG) and immunoglobin M antibody (IgM)) and laboratory data (i.e., complete blood count (CBC) with platelet count). The inclusion of family DF history was to investigate whether a previous diagnosis or history of DF in a family is associated with increased depressive and anxiety symptoms scores among the patients with DF. Clinical DF diagnosis was made under the following circumstances in three categories: probable (lived or traveled to endemic areas, has fever, and two DF symptoms); dengue hemorrhagic fever (DHF) with or without warning signs (abdominal pain, vomiting, mucosal bleeding, lethargy, liver enlargement (>2 cm), decreased platelet count or thrombocytopenia); and severe dengue (severe plasma leakage, hemorrhage, and organ impairment) [1]. These were used to identify the patient’s current dengue infection phase (acute, febrile–critical, and recovery phase). Acute phase is the onset of high-grade fever and other warning signs (e.g., headache, myalgia, arthralgia, nausea and vomiting, mucosal bleeding, and thrombocytopenia) usually lasting for a week. Some patients may develop rashes, signaling the beginning of the recovery phase [1]. During this phase, well-being improves, some warning signs tend to abate, and platelet count typically starts to rise [1]. Clinical symptoms or warning signs were also queried.

#### 2.4.2. Response Variables

The Revised Child Anxiety and Depression Scale (RCADS) is an assessment tool for screening depressive and anxiety symptoms in children and adolescents [39]. We used the RCADS-25 (25 items), the shorter version of RCADS-47 developed by Chorpita et al. [39], with child and parent versions [40]. The child version was administered to pediatric patients and controls, whereas the parent version was administered to parents of pediatric patients who could not complete the child version due to sickness and other comorbidities. RCADS-25 has two scales: depression (10 items) and anxiety (15 items); items were rated on a 4-point Likert-scale from 0 (“never”) to 3 (“always”) and were scored and interpreted using a “*t*-score” [40]. T-scores are transformed raw scores used to obtain a normal distribution with a maximum score of 80. A *t*-score of 65 means borderline and a *t*-score of 70 means clinical threshold [40]. The *t*-scores were adjusted for gender (girl and boy) and grade level (grade 3 to 12); the child version has different *t*-scores from the parent version [40]. Both depression and anxiety scales have good internal consistency (Cronbach’s alpha) of α = 0.78 and α = 0.88 in clinical and general or school-based populations [37,38,39,40,41]. This tool has been reported to be a cross-culturally reliable measure of depressive and anxiety symptoms for children and adolescents [42].

Both RCADS-25 child and parent forms were translated to Filipino (Tagalog) by independent bilingual translators and was then face, content, and construct validated by experts [43]. Then it was pilot tested among selected clinical and school-based samples. When a consensus was reached, it was translated back to English by bilingual translators. We calculated the internal consistency (Cronbach’s alpha) of the RCADS-25 child and parent forms in Filipino, and results evidenced an acceptable internal consistency which ranged from α = 0.70 to α = 0.80 in clinical samples and α = 0.73 to α = 0.83 in school-based samples, but were not included in the final analysis.

RCADS-25 was used to screen the presence of depressive or anxiety disorder symptoms and not to diagnose disorders associated with the mentioned symptoms. Thus, we recommended further psychiatric assessment among patients whose scores suggested the presence of borderline or clinical levels of depressive and anxiety symptoms.

Self/parent-reported psychiatric manifestations: Patients and their parents/guardians were also asked to report other psychiatric manifestations that they observed during the onset of the disease and hospitalization through a structured interview based on the diagnostic criteria of depressive and anxiety disorders of the Diagnostic and Statistical Manual of Mental Disorders 5th Edition (DSM-V) [44]. Similar to the results of the RCADS-25, patients who reported the presence of psychiatric symptoms were also recommended for further psychiatric assessment.

### 2.5. Statistical and Data Analysis

Statistical analyses were conducted using the Statistical Package for Social Sciences version 25 (IBM Corp., Armonk, NY, USA). The Chi-squared test was used to test the significant differences between the groups in terms of their socio-demographic profile and in the prevalence (*%*) of pediatric patients and controls with borderline or clinical depressive and anxiety symptoms. Odds ratio effect sizes were also computed to measure the differences in the proportion of those with depressive and anxiety symptoms among pediatric and controls. An independent *t*-test (2-tailed) was also conducted to test the significant differences in the mean depression and anxiety scores (*t*-scores) between the two groups. We also reported the effect sizes of the mean differences between the two groups in depressive and anxiety symptoms by calculating Cohen’s *d*, Glass’ *delta*, and Hedges’ *g* effect size estimations. Multiple linear regression was done by inputting all variables (dummy variables (i.e., 0 or 1) for categorical variables) in the model using a stepwise method in forward selection to identify significant (*p* < 0.05) predictors of depressive and anxiety symptoms. Patients’ reported psychiatric manifestations were translated into English by an independent bilingual translator and were analyzed using content analysis, a method that can identify patterns across qualitative data (words or phrases) that can be counted (frequency) for quantitative analyses [45,46,47].

## 3. Results

### 3.1. Socio-Demographic Profile, Clinical Parameters, and Symptoms

Initially, 625 participants (pediatric patients *n* = 321; controls *n* = 304) were recruited in the study, but only 485 (pediatric patients *n* = 225; controls *n* = 260) were found eligible, complied to the informed consent procedures, and participated in this study. Among the 225 data collected from the patients with DF, 155 (68.9%) were from pediatric patients with DF and 70 (31.1%) were from parents of other patients with DF who could not answer the screening tool. Similarly, the 260 data collected among controls were from 220 (84.6%) students and 40 (15.4%) parents of children who had the same age and grade level as the patients with DF. The profile of the participants is shown in Table 1.

The two groups had significantly different proportions and distributions in terms of their age, education, income, and households. In terms of gender, the two groups were significantly the same, where both had nearly the same distribution (50%) of each gender. Pediatric patients had a mean age (M) of 10.96 and a standard deviation (±) of 2.95 years, 53% were males, the majority had DHF with warning signs (76.4%) and were in the acute (febrile–critical) phase of DF (79.6%) of the infection. Thrombocytopenia (low platelet counts) and petechiae (rashes) were the most commonly reported symptoms among the patients. The controls, male (*n* = 133) and female (*n* = 127), had a mean age of 12 (±2.8) years.

### 3.2. Prevalence and Mean Score Differences of Depressive and Anxiety Symptoms

More pediatric patients with DF (13.3%) had borderline or clinical depressive symptoms than controls (3.5%). Similarly, a significant proportion (34.2%) of pediatric patients with DF compared with controls (*n* = 42; 16.2%) had borderline or clinical anxiety symptoms. Chi-squared analyses also revealed that pediatric patients with DF had a significantly (*p* ≤ 0.001) higher prevalence of depressive and anxiety symptoms than the controls, as shown in Table 2. This represents the fact that based on the odds ratio, pediatric patients were 4.3 times more likely to have depressive symptoms and 2.7 times more likely to have anxiety symptoms than controls. Moreover, when we compared the mean scores of the participants, pediatric patients had significantly (*p* ≤ 0.001) higher mean depressive and anxiety mean scores than controls (also shown in Table 2). The significant effects in the differences between the mean scores also represented a fairly substantial medium effect sized estimations in depression (r = 0.30–0.32) and anxiety (r = 0.30–0.34) between the two groups based on Cohen’s *d*, Gates’ *delta*, and Hedges’ *g* effect size estimations.

### 3.3. Predictors of Depressive and Anxiety Symptoms

The multiple linear regression analysis (Table 3) showed significant (*p* < 0.001) regression models in depressive and anxiety symptoms. Although the coefficients (R^2^) were low (<1), significant predictors like the presence of myalgias and arthralgias and a family history of DF were found to increase the depressive symptoms score, whereas ≤2 days of hospitalization and age (older) both increased anxiety symptom scores among pediatric patients with DF.

### 3.4. Self/Parent-Reported Psychiatric Manifestations

Table 4 shows the results of content analysis on the reported psychiatric manifestations during the onset of infection. Our quantitative analysis shows that 60 (26.7%) pediatric patients with DF had irritable mood or irritability, agitation, visual hallucinations, and aggressiveness during the onset of dengue infection. However, the reports of these manifestations were not sufficiently intense and not clinically assessed by a psychiatrist (patients’ unwillingness to be referred) to qualify for an anxiety disorder or depressive disorder due to the span (less than six months) of time these symptoms were present among the patients.

## 4. Discussion

Results show that the prevalence of depressive (13.3%) and anxiety (34.2%) symptoms among pediatric patients with DF was significantly (*p* ≤ 0.001) higher than that among controls (3.5% and 16.2%, respectively). Similarly, analysis of mean scores also showed that pediatric patients had significantly (*p* ≤ 0.001) higher depressive and anxiety mean scores than controls. The regression model identified that the presence of myalgias and arthralgias, family history of DF, ≤2 days of hospitalization, and age (older) were significant (*p* ≤ 0.001) predictors of increased depressive and anxiety symptoms among pediatric patients with DF. Additionally, 26.7% of pediatric patients with DF also reported psychiatric manifestations like irritability, agitation, visual hallucinations, and aggressiveness during the onset of the infection.

To our knowledge, this study is one of the very first reports to measure the prevalence of depressive and anxiety symptoms among patients with DF; more so, among pediatric patients with DF compared it to controls with the use of a standardized self-report screening tool. The study also provided an initial investigation on the predictors of anxiety and depressive symptoms among pediatric patients with DF by including clinical data (e.g., DF history, serologic test results, DF symptoms). This study also extends previous quantitative and qualitative (case studies) studies by using a standardized screening tool and structured interview in collecting information on the presence of psychiatric manifestations during the onset of infection. Thus, the combination of these quantitative and qualitative techniques was also considered as one of the strengths of this study.

The difference between the prevalence of depressive and anxiety symptoms between pediatric patients and controls may have been brought about by the presence of pain related to myalgia and arthralgia which was one of the predictors of increased depressive symptom scores. The presence of these symptoms has been reported to be the result of inflammatory cytokines, such as Interleukin-1 beta (IL-1 β), Interleukin-2 (IL-2), and Interleukin-6 (IL-6) [48]. These cytokines were found to be of significantly higher levels among children and adolescents with major depressive disorder (MDD) compared with healthy controls [49,50,51,52]. Thus, we propose that the presence of these cytokines, which were absent among controls, may explain the possible presence and increased levels of depressive symptoms among pediatric patients during DF infection. 

Predictors like ≤2 days of hospitalization and family history of DF, may also explain the increased depressive mood and anxiety symptoms among pediatric patients compared to that of controls. High depressive and anxiety symptoms during the ≤2 days of hospitalization would have been aggravated by the pediatric patients’ fear of medical settings and receiving medical care as common symptoms of anxiety [53]. Children usually report medical procedures, such as drawing of blood, as traumatic and seeing medical professionals as traumatic as well [54]. Further, family history of DF also tended to increase depressive symptoms among pediatric patients with DF. An indicator of anxiety disorders in children is worrying about multiple issues like future events [55], for instance, contracting DF. This can be linked to a painful past experience, such as a family member diagnosed with, and hospitalized due to, DF. Observing the severity and seriousness of symptoms, the level of associated pain may have affected patients’ perception of the disease and thus their response may have presented as depressive symptoms. Unlike controls, pediatric patients with DF were anxious of hospital conditions like limited food options, limited physical activities, limited time playing with friends, and being absent in school [56,57].

Opposite to the results of our previous study which found that as the age of pediatric patients with DF increased, their attitude towards DF decreased [58], this present study found that as their age increased, their anxiety also increased. Thus, their knowledge about their disease, rather than their attitude towards it, may explain this finding. It has been reported that children and adolescents with chronic illnesses have limited knowledge of their conditions and perceive their diseases differently from a much older population [59,60]. Thus, older children who have more of an ability to correctly understand the meaning of their disease and its severity, had higher anxiety symptoms than the younger ones.

Our study also extends previous studies which reported adult patients with DF had auditory and visual hallucinations, agitation and delusional fears, irritable affect or behavior, persecutory delusions, and psychotic episodes during DF infection [7,12]. Our results confirm that pediatric patients with DF also exhibit psychiatric manifestations during DF infection. Although these manifestations were not qualified to be considered as depressive and anxiety disorder symptoms due to the duration of their presence, it still confirms that these symptoms also impact children with DF. We found out that 26.7% of pediatric patients with DF reported irritable mood/irritability, visual hallucination, agitation, and aggressiveness. Children are more likely to be irritable, agitated and report possible delusions or hallucinations which are symptoms of MDD [53,61], than older patients. Irritability which is an overlapping symptom among children and adolescents [55], is also a diagnostic criterion for anxiety disorder, for MDD and for disruptive mood dysregulation disorder of the new DSM-V and oppositional defiant disorder (ODD) of the International Classification of Disorders (ICD-11) among children [62]. Further, hallucinations are key features of psychotic conditions and acute neurological states, such as encephalitis, and are often presented with delirium and delusions which are more prevalent in children than adults [63]. Since these results were not confirmed by a psychiatric assessment, studies that include clinical assessment by a psychiatrist and evaluation through imaging techniques may help shed light on whether these symptoms are associated with DF infection virus or not.

The low consent rate (<1%) for referral to further psychiatric assessment to assess the false positive and negative responses of the patients in the screening tool and reported psychiatric symptoms, has highlighted how patients view mental health disorders. This may be affected by cultural orientation and perception of depressive and anxiety symptoms and treatment-seeking response among patients with DF in this study. In the Philippines for example, as a collectivist-oriented culture [64], with greater social support and where family and wider society are more highly considered than one’s own self, a lower tendency to develop depressive disorder symptoms can be expected [65]. Moreover, many cultures view mood disorders as a social and moral problem associated with stigma and loss of reputation, not as mental health problems [66]. These conditions are perceived as a moral or character development that requires self-mastery and endurance and help or treatment with medication is seen as unnecessary [67].

We also identified a number of limitations in our methods: failure to match cases with controls, income and hospital setting, time of collection and lack of further psychiatric assessment by a psychiatrist. First, we failed to match pediatric patients with DF and controls at least by age which decreased the ability of our study to draw generalizable conclusions from the findings. As we mentioned, matching cases and controls was difficult due to the limited number of eligible controls. Second, the inclusion of public tertiary hospitals limited the study findings to low-income families, thus, future studies may include patients admitted to private hospitals to see whether income and hospital setting are confounding the relationship between depressive and anxiety symptoms and DF infection. Third, this study is limited only to DF cases during the peak transmission or rainy seasons from July to November. Thus, the results that patients with DF had high depressive and anxiety symptoms during this time may not apply to other periods where there is low transmission of DF. Future studies may rely in comparing patients interviewed during the high transmission season and patients interviewed during low transmission season in order to investigate whether results collected may be generalizable in any period of time and not due to the panic and alarm brought by a large number of DF cases. The fourth and the most important, was the lack of further psychiatric assessment by a psychiatrist to confirm the scores (false positive responses) in RCADS-25 and the reported psychiatric manifestations. Other than the scores in RCADS-25, we had no other means to encourage them to seek further psychiatric assessment. 

## 5. Conclusions

Our study highlights that there was a high prevalence of depressive and anxiety symptoms among pediatric patients with DF compared with controls, due to social and clinical factors which might be associated with DF infection. We also learned that pediatric patients with DF also exhibit psychiatric manifestations such as irritable mood/irritability, visual hallucination, agitation, and aggressiveness like adult patients with DF which has been reported in previous studies. Thus, it is important to screen patients with DF for these psychiatric symptoms, and if necessary, healthcare professionals must refer and encourage them to seek help to avoid long-term post-DF chronic psychiatric complications in the future. However, information on whether these symptoms are present only during the infection and may disappear on their own or may still persist after the infection, is unknown. Thus, longitudinal post-DF recovery studies would provide information on the possibilities that these symptoms may or may not develop to subsequent chronic psychiatric conditions in the future. Most importantly, this study provides benchmark information on the possible causal or direct link between depressive and anxiety symptoms and DF infection among pediatric patients with DF, yet, there is insufficient evidence to draw conclusions. Thus, the potential trauma of hospitalization and not directly DF infection, may have caused the increased depressive and anxiety symptoms among the pediatric patients with DF. Therefore, future studies must distinguish between children’s adverse reactions to hospitalization and psychiatric symptoms due to DF. While RCADS-25 is widely used and psychometrically salient, the lack of further psychiatric assessment among patients with borderline or clinical depressive and anxiety symptoms and who reported the presence of other psychiatric symptoms, hindered us to conclude whether these symptoms were directly due to DF infection or not. More so, determining whether the findings may only be specific to pediatric patients with DF infection or may be extended to children with other serious infectious diseases in general, also remains unknown and needs further studies.

## Figures and Tables

**Table 1:** Socio-demographics, clinical parameters, and clinical symptoms of pediatric patients with dengue fever (DF) and controls.

Socio-Demographic Profile		Patients	Controls	*X* ^2^
		*n* = 225	*n* = 260	*p*-Value
Gender	Male	120 (53.3)	133 (51.2)	0.65
	Female	105 (46.7)	127 (48.8)	
Age	8–11	71 (31.6)	132 (50.8)	<0.001
	12–14	67 (29.8)	61 (23.5)	
	15–17	87 (38.7)	67 (25.8)	
Education	Grade School	78 (35.6)	132 (50.8)	0.004
	Junior HS	106 (48.4)	94 (36.2)	
	Senior HS	35 (16.0)	34 (13.1)	
Income (₱)	≤10,000 PHP	171 (83.4)	21 (9.0)	<0.001
	≥11,000 PHP	34 (16.6)	213 (91.0)	
Household size	≤5 members	110 (53.12)	209 (88.6)	<0.001
	≥6 members	97 (46.9)	27 (11.4)	
Clinical Parameters				
Medical diagnosis	Clinical	24 (10.7)		
	Probable	23 (10.2)		
	DHF w/ws	172 (76.4)		
	Severe dengue	6 (2.67)		
Days in the hospital	≤2 days	150 (66.7)		
	≥3 days	46 (20.4)		
DF history	Had DF	11 (4.89)		
	First-time	185 (82.2)		
Family DF history	None	135 (60.0)		
	≥1 member/s had DF	59 (26.2)		
Dengue phase	Acute	179 (79.6)		
	Recovery	46 (20.4)		
Dengue tests	(−) NS1Ag	59 (26.2)		
	(+) NS1Ag	39 (17.3)		
	(−) IgG	21 (9.33)		
	(+) IgG	39 (17.3)		
	(−) IgM	48 (21.3)		
	(+) IgM	12 (5.33)		
Clinical Symptoms				
Headache	Symptomatic	35 (15.6)		
Fever	Symptomatic	38 (16.9)		
Nausea and vomiting	Symptomatic	55 (24.4)		
Retro-/peri-orbital pain	Symptomatic	16 (7.11)		
Myalgias & arthralgias	Symptomatic	48 (21.3)		
Abdominal pain	Symptomatic	87 (38.7)		
Petechiae (rash)	Symptomatic	96 (42.7)		
Thrombocytopenia	≤9900/mm^3^	143 (63.5)		

DF—dengue fever; HS—high school; ₱—Philippine peso (52.16 USD = 1 ₱); Acute—febrile to critical phase; ws—warning signs; DHF—dengue hemorrhagic fever; (+)—positive; (−)—negative; NS1Ag—non-structural protein 1 antigen; IgG—immunoglobin G antibody; IgM—immunoglobin M antibody.

**Table 2:** Prevalence and mean score differences of depressive and anxiety symptoms between pediatric patients with DF and controls.

Symptoms	Prevalence			Mean Score Difference		
	Patients (*n* = 225)	Controls (*n* = 260)	*X* ^2^	Patients (*n* = 225)	Controls (*n* = 260)	*t*-Test (2-Tailed)
	*n* (%)	*n* (%)	*p*-Value	Mean (SD)	Mean (SD)	*p*-Value
Depressive symptoms	30 (13.3)	9 (3.5)	<0.001	52.3 (9.87)	49.3 (8.64)	<0.001
Anxiety symptoms	77 (34.2)	42 (16.2)	<0.001	59.3 (11.5	55.8 (9.34)	<0.001

*x*^2^—Chi-squared; SD—standard deviation.

**Table 3:** Predictors of depressive and anxiety symptoms among pediatric patients with DF.

Outcome Variables	Predictors	*R* ^2^	*β*	Predictor *p*-Value	Model *p*-Value
Depressive symptoms	Myalgias and arthralgias	0.07	4.87	0.002	<0.001
	Family history of DF		3.19	0.028	
Anxiety symptoms	Age	0.15	1.26	<0.001	<0.001
	Days in the hospital (≤2 days)		1.26	0.005	

β—beta coefficients; DF—dengue fever.

**Table 4:** Self/parent-reported psychiatric manifestations among pediatric patients with DF using content analysis and quantitative analysis.

Manifestations (In Filipino) *	English Translation/Meaning	Patterns (DSM-V) ^a^	Pediatric Patients (*n* = 60)
Mainit ang ulo	Irritable mood	Irritable mood	24 (40.0)
Mainitin ang ulo	Irritable		
Irritable	Irritable	Irritability	12 (20.0)
Naiirita	Irritable		
Bugnutin	Quick-tempered		
Worry	Worry	Worry	2 (3.3)
Kinakabahan o natatakot	Nervous/fear that something awful might happen		
Nag-Hallucinate noong nilalagnat	Hallucination	Hallucination	4 (6.7)
Isip-bata	Childish		
Hindi kami kilala; iba ang nakikita niyang mukha noong nilalagnat	Visual hallucination		
Madalis magalit	Easily-angered/agitated	Agitation	9 (15.0)
Pikon	Easily angered by jokes or jests, touchy		
Masungit	Ill-tempered, short-tempered/agitated		
Aggressive	Aggressive	Aggression	4 (6.7)
Sumisigaw	Shouting		
Hindi Nagsasalita	Does not talk	Quiet	2 (3.3)
Tahimik	Quiet		
Tinatamad	Laziness	Fatigue	2 (3.3)
Matamlay	Listless: seeming too tired to care about anything, not interested in things, not caring to be active		
Nanghihina	Feeling weak		
Mahirap kausap	Hard to talk with	Hard to talk with	1 (1.67)

* Responses in Filipino (Tagalog) language were analyzed and translated to English by an independent bilingual translator; ^a^ based on the Diagnostic and Statistical Manual of Mental Disorders 5th Edition (DSM-V) diagnostic criteria for depressive and anxiety disorders.

## Supplementary Materials

The following are available online at https://www.mdpi.com/1660-4601/17/1/99/s1. Supplementary File S1: STROBE statement.
